# Pleiotropic effects of alpha-SNAP M105I mutation on oocyte biology: ultrastructural and cellular changes that adversely affect female fertility in mice

**DOI:** 10.1038/s41598-019-53574-8

**Published:** 2019-11-22

**Authors:** Matilde de Paola, María Paz Miró, Marcelo Ratto, Luis Federico Bátiz, Marcela Alejandra Michaut

**Affiliations:** 1 Instituto de Histología y Embriología, Universidad Nacional de Cuyo-CONICET, Mendoza, Argentina; 2 Facultad de Ciencias Médicas, Universidad Nacional de Cuyo, Mendoza, Argentina; 3 Instituto de Medicina y Biología Experimental de Cuyo, Universidad Nacional de Cuyo-CONICET, Mendoza, Argentina; 4 Instituto de Anatomía, Histología y Patología, Facultad de Medicina, Universidad Austral de Chile, Valdivia, Chile; 5 Instituto de Ciencia Animal, Facultad de Ciencias Veterinarias, Universidad Austral de Chile, Valdivia, Chile; 6 Centro de Investigación e Innovación Biomédica (CIIB), Facultad de Medicina, Universidad de los Andes, Santiago, Chile; 7 Facultad de Ciencias Exactas y Naturales, Universidad Nacional de Cuyo, Mendoza, Argentina

**Keywords:** Cell biology, Medical research

## Abstract

After sperm-oocyte fusion, cortical granules (CGs) located in oocyte cortex undergo exocytosis and their content is released into the perivitelline space to avoid polyspermy. Thus, cortical granule exocytosis (CGE) is a key process for fertilization success. We have demonstrated that alpha-SNAP -and its functional partner NSF- mediate fusion of CGs with the plasma membrane in mouse oocytes. Here, we examined at cellular and ultrastructural level oocytes from hyh (hydrocephalus with hop gait) mice, which present a missense mutation in the Napa gene that results in the substitution of methionine for isoleucine at position 105 (M105I) of alpha-SNAP. Mutated alpha-SNAP was mislocalized in hyh oocytes while NSF expression increased during oocyte maturation. Staining of CGs showed that 9.8% of hyh oocytes had abnormal localization of CGs and oval shape. Functional tests showed that CGE was impaired in hyh oocytes. Interestingly, *in vitro* fertilization assays showed a decreased fertilization rate for hyh oocytes. Furthermore, fertilized hyh oocytes presented an increased polyspermy rate compared to wild type ones. At ultrastructural level, hyh oocytes showed small mitochondria and a striking accumulation and secretion of degradative structures. Our findings demonstrate the negative effects of alpha-SNAP M105 mutation on oocyte biology and further confirm the relevance of alpha-SNAP in female fertility.

## Introduction

Fertilization is a fundamental and highly orchestrated process that involves the fusion of two haploid gametes, the sperm and the oocyte, to create a unique diploid individual. Besides contributing with half of the nuclear genetic material, the oocyte provides the embryo with almost all membrane and cytoplasmic components that are required for successful fertilization and early embryogenesis^1^. Unfertilized mature oocytes of many invertebrates and vertebrates contain membrane-bound secretory organelles located in the cortex, known as cortical granules (CGs; reviewed by Liu M, 2011^2^). CGs are originated from the Golgi complex and they accumulate all over the cytoplasm during oogenesis^2^. During oocyte maturation, CGs migrate to the cortex -the area just under the oocyte’s plasma membrane- by two different mechanisms^3^ and anchore to the cortical actin network^4^. In rodents, there is a particular cortical organization with an area devoided of CGs associated to the meiotic spindle known as cortical granule free domain (CGFD)^5^. Interestingly, the presence of CGFD seems to be exclusive of rodents oocytes since porcine, feline, bovine, equine and human oocytes lack such domain^6–11^. Thus, CGs and CGFD are two areas well established in mouse eggs determined by the assymetry of cortical granule localization^12,13^.

Upon gamete fusion, the fertilized oocyte needs to prevent fertilization by more than one sperm, a condition known as polyspermy. Although physiological polyspermy occurs in numerous species including birds, reptiles and insects^14^, in mammals it is considered an abnormal phenomenon because it would result in severe developmental failures of the embryo^15^. In order to prevent polyspermy, the fusion of the first sperm (which occurs only in CGs area in mouse oocyte) induces the exocytosis of CGs, also known as cortical reaction, and the content of granules is released into the perivitelline space. Consequently the membrane and zona pellucida are biochemically altered, becoming the oocyte unreceptive to an additional sperm fusion^16^ and providing a definitive block to polyspermy^17,18^.

The signal transduction pathway accountable for cortical reaction is not yet completely understood and is thought to be mediated by SNARE (Soluble N-ethylmaleimide Sensitive Factor [NSF] Attachment Protein [SNAP] Receptor) proteins: SNAP-25, Syntaxin and VAMP, which are key elements of the membrane fusion machinery. In fact, in mouse oocytes, two proteins of this machinery have been characterized: SNAP-25^19^ and Syntaxin 4^20^, and more recently, SNAP-23 has also been involved^21^. In porcine oocytes, it has been documented that the SNAREs: Syntaxin 2, SNAP23, VAMP1, and VAMP2 are involved in cortical reaction^22^. In neurons, SNARE complexes bring the vesicle and plasma membranes together (trans-SNARE) leading to membrane fusion. After membrane fusion, SNARE complexes remain on the same membrane (cis-SNARE) and are disassembled by the concerted action of N-ethylmaleimide-sensitive factor (NSF) and soluble NSF attachment proteins (SNAPs) in a postfusion step to recycle the SNAREs for another round of fusion^23^. Nevertheless, we have previoulsy described that in mammalian gametes, alpha-SNAP and NSF participate in a prefusion step^24,25^.

Neuronal exocytosis of synaptic vesicles is rapid (in the order of miliseconds and seconds) and vesicles are recycled after the release of their contents^26^. Neither of these characteristics is true for cortical granule exocytosis in mammalian eggs. On the contrary, cortical granule exocytosis is a slow process (in the order of minutes) -as we have previously demonstrated- and cortical granules are not recycled^27^. Assembly of the SNARE core complex during cortical granule exocytosis is triggered by cues that are only partially known. We have documented that alpha-SNAP and NSF participate in cortical granule exocytosis and proposed a working model in which the alpha-SNAP/NSF complex disassemble cis-SNARE in a prefusion step^24^. Once the tripartite core complex is assembled, it promotes fusion of the cortical granules with plasma membranes leading to the secretion of cortical granule’s content. Hence, defects in SNARE complex formation –including SNARE primings- could be one of the causes of polispermy and female infertility.

Despite the widespread acceptance of this model, SNARE complex disassembly is not the only function of SNAPs and NSF^28^. In fact, alpha-SNAP has been implicated in novel SNARE/NSF-independent functions in different cellular processes such as autophagy^29,30^; apoptosis^31–33^; cell to cell contact^34,35^, adhesion to the extracellular matrix and cell motility^36^; AMPK signaling, mitochondrial biogenesis^37^ and store-operated calcium entry^38,39^.

A recessive inheritable disease known as hydrocephaly with hop gait (hyh) in mice is caused by a spontaneous G → A missense mutation in exon 4 of Napa (N-ethylmaleimide- sensitive factor attachment protein, alpha) gene. This point mutation resulted in a substitution of a highly conserved methionine for isoleucine at aminoacid residue 105 (M105I)^40,41^. Because deletion of alpha-SNAP causes early embryonic lethality in mice^40^, hyh mice provide an excellent *in vivo* model to characterize the role of alpha-SNAP M105I in fertility from a systems biology perspective.

We have reported that M105I mutation in hyh females plays a critical role in the balance between folliculogenesis and follicular atresia producing a reduced ovulation rate and, consequently, a noticeable reduction of female fertility^42^. In this work, we aimed to investigate the consequence of mutated alpha-SNAP in the female gamete. We found that M105I mutation in alpha-SNAP leads to a dramatic decrease in fertility due to severe multifactorial defects at cellular and ultrastructural level in hyh oocytes.

## Results

### alpha-SNAP M105I is mislocalized in hyh oocytes

**Figure 1:**
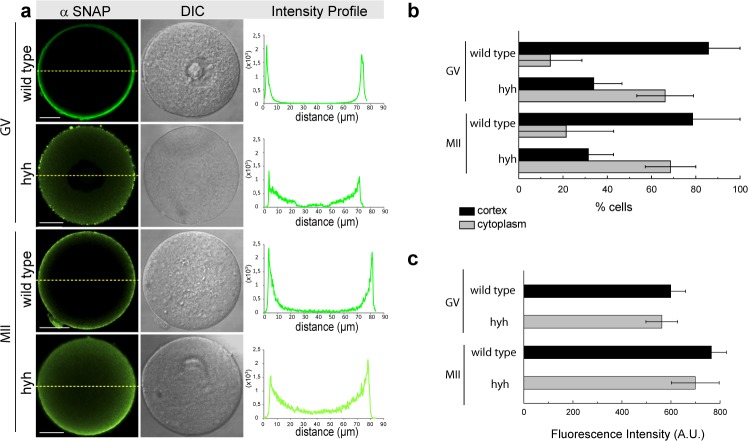
Alpha-SNAP localization during meiotic maturation in wild type and hyh oocytes. (**a**) Alpha-SNAP was immunodetected at two different stages during meiotic maturation: Germinal vesicle-intact oocytes (GV) and Metaphase II oocytes (MII). Green indicates positive staining for primary alpha-SNAP antibody detected by a secondary antibody conjugated with DyLight 488; DIC shows differential interference contrast (DIC) images. The right column shows the fluorescence intensity profiles for alpha-SNAP. Fluorescence intensities were measured along dashed yellow lines traced in each oocyte. The intensity of alpha- SNAP is indicated by green lines. Scale bar: 20 μm. (**b**) Cortical and cytoplasmic patterns of protein distribution. The cortical region was defined as the region of 10 μm thickness from the oocyte plasma membrane towards the oocyte centre. Those cells in which fluorescence decay at 10 μm were considered to present a cortical staining, and those cells which present high fluorescence at 10 μm towards the center and beyond were considered to present cytoplasmic staining. Percentage analysis was assessed. Number of oocytes: GV wild type = 33; GV hyh = 34; MII wild type = 27; MII hyh = 12. (**c**) Histogram showing fluorescence intensity quantification (A.U.: arbitrary units) for wild type GV and MII oocytes compared to hyh ones.

**Figure 2:**
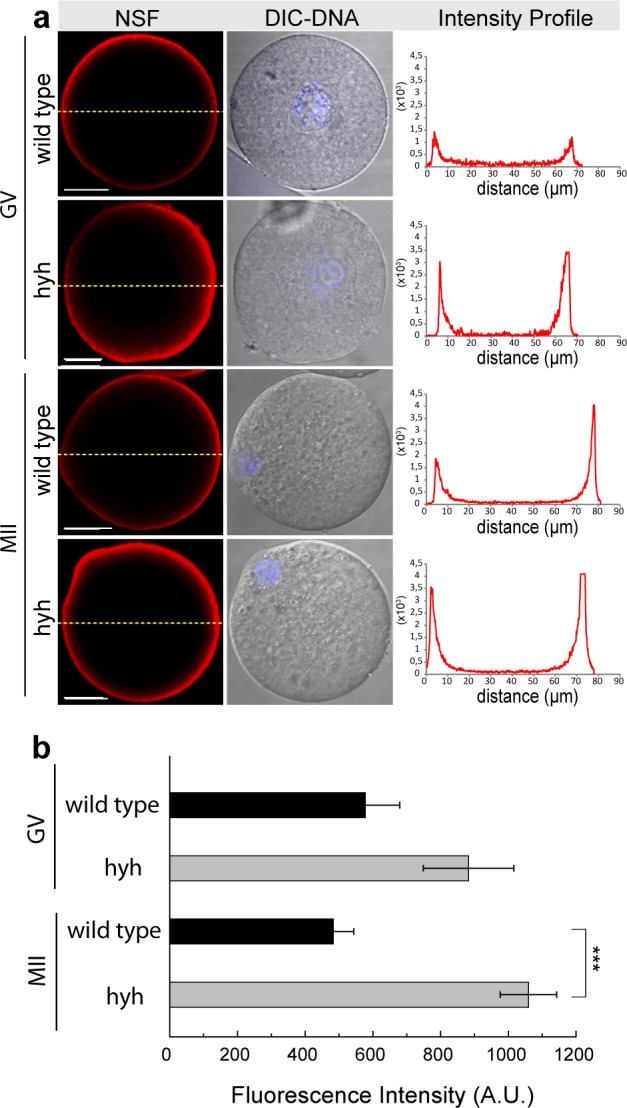
NSF inmunodetection during meiotic maturation in wild type and hyh MII oocytes. (**a**) NSF was detected by indirect immunoflurescence in wild type and mutant homozygous (hyh) GV-intact oocytes (GV) and MII oocytes (MII). Red indicates positive staining for primary NSF antibody detected by a secondary antibody conjugated to Alexa Fluor 594; blue indicates DNA, labeled with Hoechst 3342; DIC shows differential interference contrast (DIC) images. Right column in each panel shows the fluorescence intensity profiles for NSF. Fluorescence intensities were measured along equatorial dashed yellow lines traced in each oocyte. The intensity of NSF is indicated by red lines. Scale bar: 20 μm. (**b**) Histogram showing fluorescence intensity quantification (A.U.: arbitrary units) for wild type GV and MII oocytes compared to hyh ones. ***p ≤ 0.001 (Student’s t-test).

Based on a previous study on hyh mice that shows that alpha SNAP M105I leads abnormal localization of several proteins in neurons^40^, we hypothesized that the mutated alpha-SNAP might have an abnormal localization or distribution in mice oocytes. To test this, alpha-SNAP and its related partner protein NSF were analyzed during oocyte maturation by immunolocalization studies. Confocal analysis of germinal vesicle (GV)-intact oocytes and metaphase II (MII) oocytes showed that in both stages, alpha-SNAP was mislocalized in hyh oocytes. While alpha-SNAP staining was mainly concentrated in the cortex region of wild type oocytes, in hyh oocytes the staining was distributed in both cortical and cytoplasmic region (Fig. 1a). This can be better seen following single-cell fluorescence intensity profile (Fig. 1a, right panel). In order to quantify alpha-SNAP distribution at the population level, an analysis of fluorescence intensity profiles was performed. As we have previously described, two staining patterns were defined: the cortical and the cytoplasmic pattern^24^. In agreement with the results shown in single-cell confocal microscopy images and intensity profiles analysis, alpha-SNAP showed a cortical localization in nearly 80% of GV-intact oocytes and MII oocytes of wild type mice. On the contrary, in hyh oocytes, alpha-SNAP was immunolocalized predominantly in the cytoplasmic region of 70% of both GV-intact and MII oocytes (Fig. 1b). Interestingly, these changes are not associated with changes in the level of alpha-SNAP. In fact, we quantified the fluorescence’s intensity in the whole cell (independently of the intracellular distribution) in GV-intact and MII oocytes and there was no difference between wild type and hyh oocytes at every analyzed stage of oocyte maturation (Fig. 1c). Then, we analyzed the localization of NSF, the partner of alpha-SNAP in membrane fusion. In a similar analysis, we found that NSF was normally localized in the cortical region of hyh GV- oocytes and hyh MII oocytes (Fig. 2a). The analysis of fluorescence intensity profiles for NSF showed no differences between wild type and hyh oocytes (Fig. 2b) at both analyzed stages of oocyte maturation. However, the fluorescence intensity in the whole cell (as an indicator of NSF expression) was higher in GV-intact oocytes and MII oocytes (Fig. 2b) indicating that NSF is more abundant in oocyte’s cortex, independently of maturation stage. These results suggest that M105I mutation in alpha-SNAP protein alters mainly the distribution of alpha-SNAP in mouse oocytes.

### Ultrastructural analysis of hyh metaphase II oocytes

**Figure 3:**
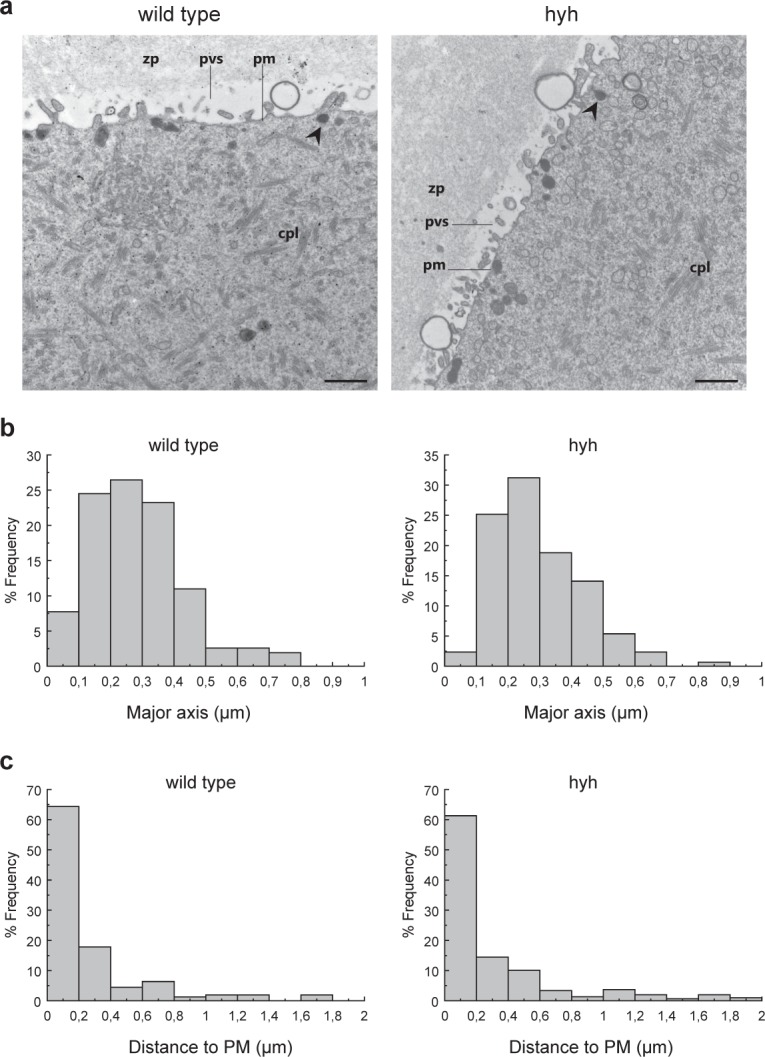
Cortical granules at the ultrastructural level in wild type and mutant hyh MII oocytes. (**a**) Representative transmission electron microscopy images of the cortex in MII oocytes (MII) in wild type and mutant homozygous (hyh) mice.Cortical granules (arrow heads) are linearly arranged below the plasma membrane. Note microvillous processes of oolema. zp: zona pellucid; pvs: perivitelline space; pm: plasma membrane; cpl: cytoplasmic lattice. Scale bar: 1 μm. Frequency histograms were plotted for cortical granules size (major axis) (**b**) and distance to the plasma membrane (**c**). Values on the x-axis represent bins of size (**a**) or distance (**b**) data. The number of analyzed cortical granules (CG) and images are the same as in Table 2. Data were compared using the Kolmogorov-Smirnov test for two sets of data. Individual size and CG distances in wild type and hyh oocytes show similar distribution profiles (N.S. p > 0.05).

Alpha-SNAP is involved in several membrane fusion events, such as intra-Golgi trafficking^43^, autophagy flux^44^, and exocytosis^45^. In addition, it has been proposed that alpha-SNAP regulates mitochondrial biogenesis in an AMPK-dependent pathway^37^. Thus, we analyzed the ultrastructure of hyh MII oocytes by transmission electron microscopy (TEM). MII oocytes from wild type and hyh superovulated female mice were collected and processed for TEM. At the ultrastructural level, mammalian cortical granules are located beneath the plasma membrane, they range in size from 0.1 μm to 1 μm in diameter with uniformly dense content, and look morphologically similar to each other^13,46,47^. We analyzed and compared morphology, size and distance to the plasma membrane for each mentioned genotype. No differences were observed in the morphology and distribution of cortical granules (CG) in wild type and hyh oocytes. As shown in Fig. 3, and in agreement with previous studies^47^, cortical granules were found in a non-docked state. Detailed quantification analysis of cortical granules size and distance to plasma membrane showed no significant differences between both genotypes (Table 1), and were similar to those reported by literature^13^. Frequency histograms analysis revealed comparable distributions of individual CG size and distances to plasma membrane, showing no significant differences between wild type and hyh oocytes (Fig. 3).

**Table 1:** Ultrastructural analysis of cortical granules in wild type and mutant homozygous (hyh) MII oocytes.

Genotype	Mean size (µm)	Mean distance to PM (µm)	N° analyzed CG	N° analyzed images
wild type	0,292 ± 0,012	0,247 ± 0,028	156	44
hyh	0,291 ± 0,008	0,304 ± 0,025	298	62

Cortical granules size (major axis) and distance to plasma membrane were evaluated only in the cortex (0–2 µm from the plasma membrane). Data are shown as mean ± SEM. For both analyzed parameters, no significant differences were found between wild type and hyh oocytes. N.S. p > 0,05 (Student’s t-test).

**Figure 4:**
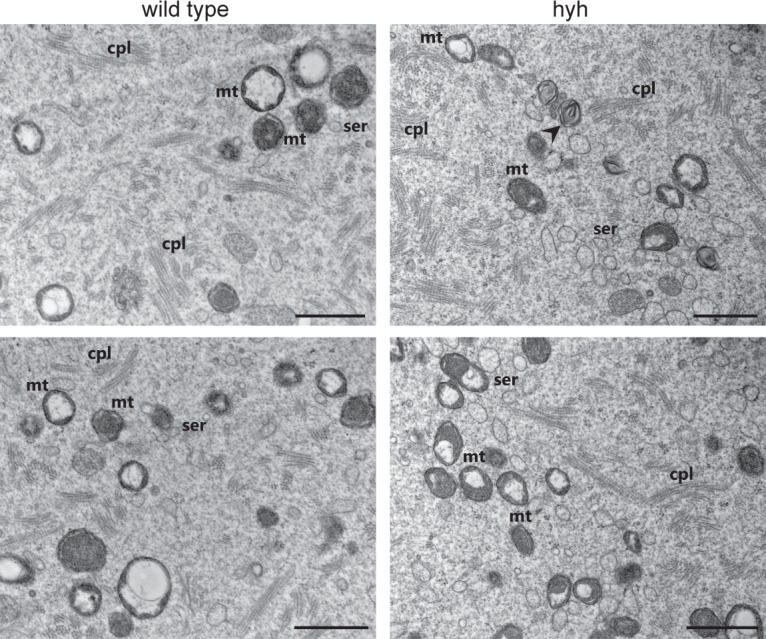
Cytoplasmic and mitochondrial ultrastructure in wild type and hyh MII oocytes. Representative electronmicrographs of MII oocytes cytoplasm from wild type and mutant homozygous (hyh) mice. Images show different cytoplasmatic structures as mitochondria, which are round or oval with or without vacuola (mt), the smooth endoplasmic reticulum (ser), which is mostly vesicular, and the extense fibrillar matrix of cytoplasmic lattice (cpl). Scale bar: 1 μm.

Mitochondria are the most prominent organelles in the oocyte cytoplasm^48^. TEM images for wild type and hyh MII oocytes showed mitochondria dispersed throughout all over cytoplasm. As expected, the mitochondrial shape was round to oval with a dense matrix and some of them presented clear vacuolated structures within their matrices (Fig. 4). While no changes were found in the shape of individual mitochondria, the percentage of vacuolated mitochondria was slightly but significantly increased in hyh MII oocytes (Table 2). As expected, the percentage of wild type vacuolated mitochondria was in accordance with levels found in superovulation treatment experiments^49^. The number of mitochondria per unit area did not differ significantly between the two groups (Table 2). However, the diameter size, usually ranging between 0.4–0.6 µm^50^, as well as vacuole size was significantly decreased in hyh compared to wild type MII oocytes (Table 2).

**Table 2:** Ultrastructural analysis of mitochondria in wild type and mutant homozygous (hyh) MII oocytes.

Genotype	Vacuolated mitochondria (%)	Mitochondria density (/µm2)	Mean mitochondria size (µm)	Mean vacuole size (µm)
wild type	43,58 ± 1,47 (1745)	0,485 ± 0,040 (48)	0,572 ± 0,007 (501)	0,380 ± 0,010 (289)
hyh	49,50 ± 1,39** (2115)	0,488 ± 0,037 N.S (58)	0,503 ± 0,006*** (524)	0,287 ± 0,007*** (257)

Percentage of vacuolated mitochondria, mitochondria density, mitochondria and mitochondrial vacuole size (major axis) were evaluated. For mitochondria density analysis, the total number of mitochondria in single TEM sections were counted, and based on the scale bar, the number of mitocondria per unit area (µm^2^) was calculated. Numbers in parenthesis indicate the number of analyzed mitochondria or analyzed sections in the case of mitochondria density. Data are shown as mean ± SEM.***p ≤ 0.001, **p ≤ 0.01, N.S. p > 0,05 (Student’s t-test).

**Figure 5:**
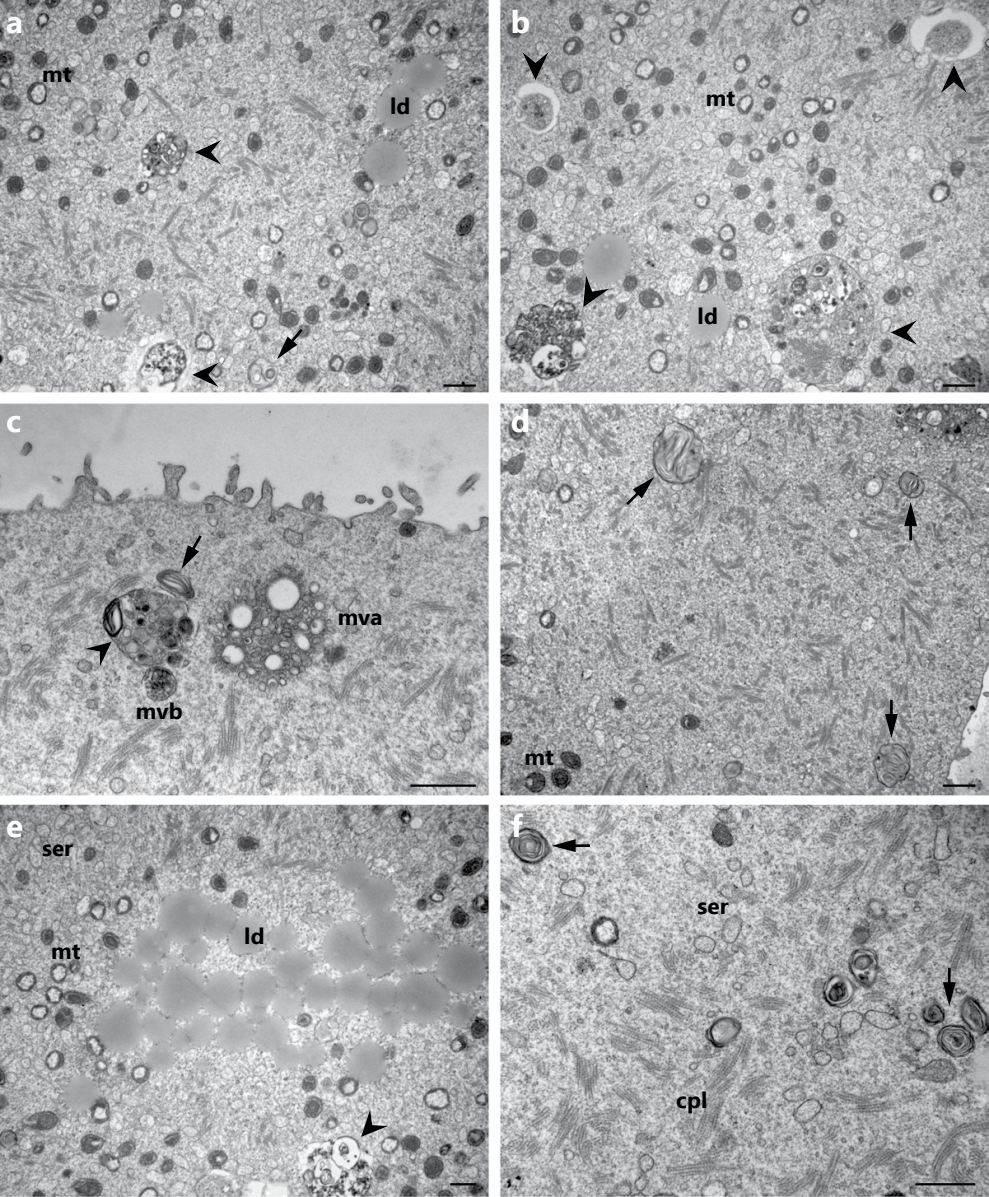
Cytoplasmic degradative structures in hyh oocytes. Representative electronmicrographs of MII oocytes from mutant homozygous (hyh) mice showing different degradative structures such as autophagic-like vesicles in different stages of maturation (arrowheads; **a**–**c**,**e**) and dense lamellar bodies (arrows; **a**,**c**,**d**,**f**). More immature autophagic-like vesicles show a double membrane separated by a wider lumen and its main contents appear to be recognizable cytoplasmic organelles. Meanwhile more mature autophagic-like vesicles are delimited by a single membrane containing membranous material of unrecognizable origin. Note in (**c**) that a multivesicular body (mvb) and a multilamellar body (arrow) are in close association with an autophagic-like vesicle (arrow head). Mt: mitochondria; ld: lipid droplet; mvb: multivesicular body; mva: multivesicular aggregate; cpl: cytoplasmic lattice; ser: smooth endoplasmic reticulum. Scale bar: 1 μm.

Strikingly, TEM observations of hyh oocytes showed a frequent presence of different structures in the oocyte cytoplasm that were morphologically compatible with an increased degradative pathway. Exclusively in hyh oocytes structures resembling a phagophore could be observed. A phagophore is a bowl-shaped double-membrane that is formed engulfing a portion of cytoplasm or other cytoplasmic components such as organelles (see arrowheads in Fig. 5b). These structures represent a clear early sign of autophagocytosis. Furthermore, enclosed membrane structures morphologically compatible with autophagic vesicles or autophagosomes, which contain remains of the cytoplasmic material and organelles, were frequently encountered in the cytoplasm of hyh oocytes (Fig. 5a–c,e). Their diverse appearances might represent different stages of evolution during autophagocytosis process^51^. The immature early autophagosomes are those where a double membrane with a marked lumen can be distinguished (Fig. 5a,b,e), which finally leads to the formation of single-membrane late autophagosomes. In these more mature autophagic vesicles the content is degraded; it becomes granular and membranous debris from which the initially included organelles cannot be recognized due to the degradative process (Fig. 5a–c). In some cases we could observe multivesicular bodies (mvb) and multivesicular aggregates (mva) in the proximity of these mature autophagosomes (Fig. 5c), demonstrating that autophagosome receives input from the endocytic pathway.

**Figure 6:**
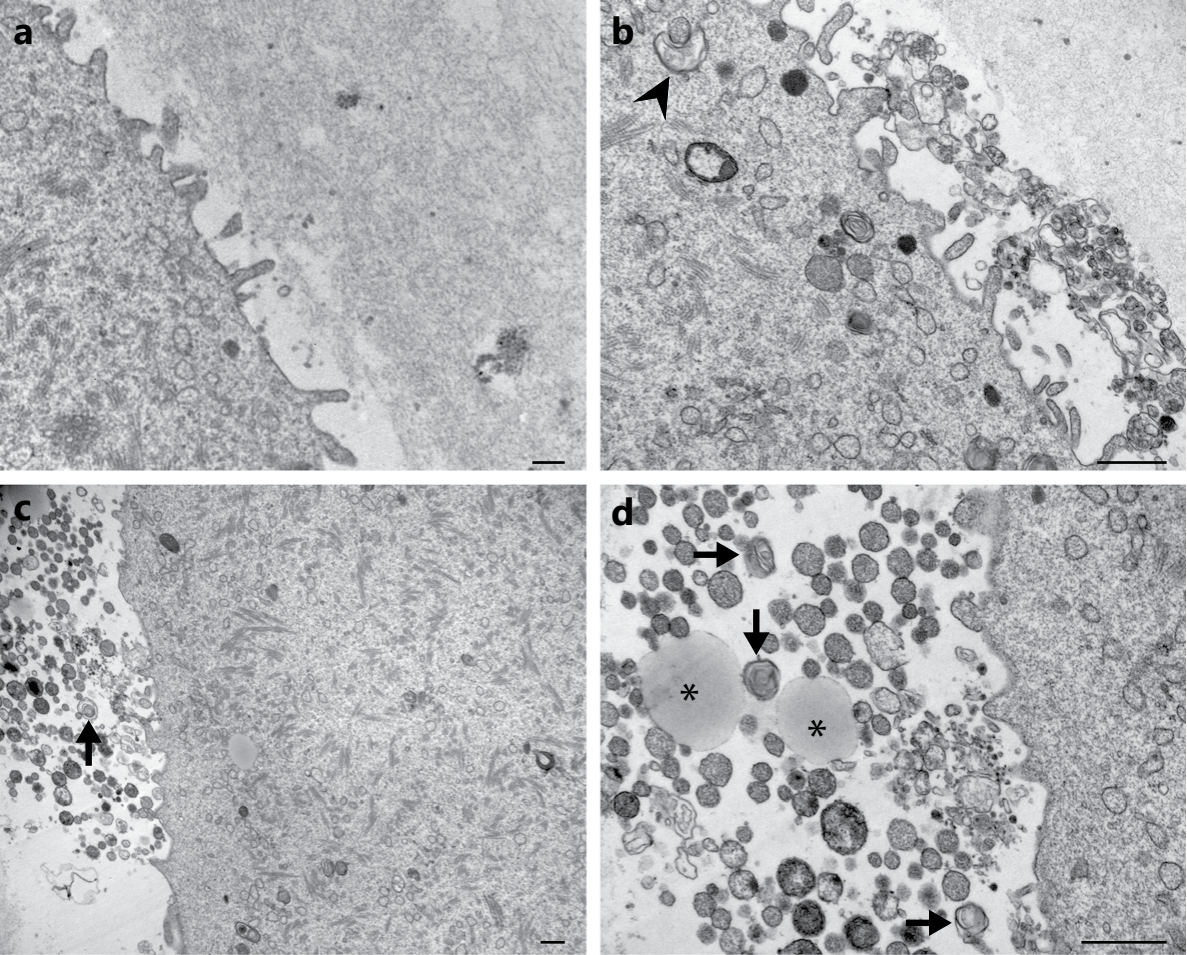
Ultrastructure of perivitelline space in wild type and hyh MII oocytes. Representative transmission electron microscopy images of perivitelline spaces from wild type (**a**) and mutant homozygous (hyh) oocytes (**b**–**d**). (**a**) Perivitelline space from wild type MII oocytes is free of extracellular structures, only oolema microvilli are observed. (**b**–**d**) Numerous residual bodies are observed in the perivitelline space of mutant homozygous (hyh) MII oocytes, many of them *a*re membrane-delimited. Additionally, remnants of cytoplasmic components as lipid droplets (asterisk) and dense lamellar bodies (arrow) are observed under higher magnification (**d**). Note phagophore-like structure surrounding a membrane bound vesicle (**b**, arrowhead). Scale bar: 1 μm.

Myelin figures, also called dense lamellar bodies, myelinated bodies or residual bodies, are whorls of indigestible membranous material frequently found in advanced stages of degradation. These structures are presumed the digestive residues of late autophagosomes, which can either be released from the cell or remain within permanently. In hyh oocytes, there was a significant increase in this type of membranous structures (Table 3). They appear dispersed throughout the cytoplasm as larger crystalline structures, or as smaller concentric whorls (see arrowhead in Figs. 4 and 5a,c,d,f). Even more striking, numerous extracellular vesicles, myelin figures, cytoplasmic remnants, and membranous debris were detected in the perivitelline space of hyh oocytes. (Fig. 6b–d). The secretion of this type of structures may be an indirect consequence of the accumulation of autophagosomes in the cytoplasm and could be consistent with residual bodies’ exocytosis or autophagic exocytosis^52^. It is important to point out that no accumulation of this type of structures was observed in the perivitelline space of wild type oocytes (Fig. 6a). These findings indicate that M105I mutation in alpha-SNAP increases – directly or indirectly- the degradative pathway in hyh oocytes.

**Table 3:** Quantification of dense lamellar bodies in wild type and mutant homozygous (hyh) MII oocytes.

Genotype	N° analyzed images	Dense lamellar bodies
wild type	113	0,5 66 ± 0,090
hyh	113	1,707 ± 0,147***

Dense lamellar bodies or myelin figures found in randomly selected TEM sections were quantitated in wild type and mutant homozygous (hyh) MII oocytes. Data are shown as mean ± SEM. ***p ≤ 0.001 (*Student’s* t-test).

Altogether, TEM findings indicated that at the ultrastructural level, there were no differences in CG between wild type and of hyh oocytes. However, hyh oocytes showed unexpected alterations in the cytoplasm and perivitelline space. In the cytoplasm, hyh MII oocytes showed the presence of small and vacuolized mitochondria and degradative vesicles. In the perivitelline space, hyh MII oocytes showed the presence of extracellular material with similar morfology to the degradative structures observed in the oocyte cytoplasm. More studies are needed to better characterize these alterations.

### Atypical distribution of cortical granules is only observed in oval hyh oocytes

**Figure 7:**
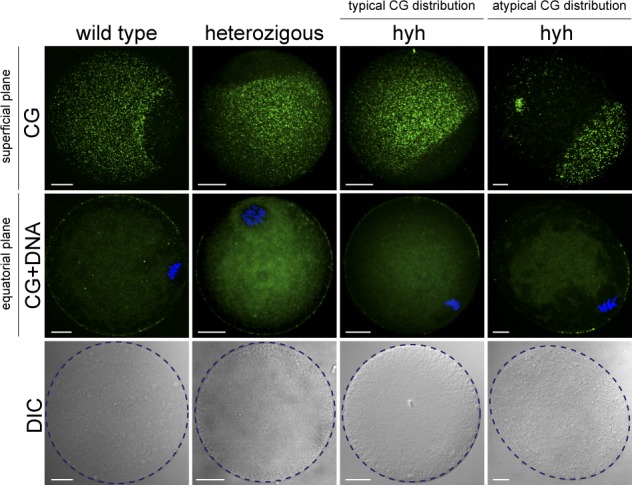
Distribution patterns of cortical granules in MII oocytes from wild type, heterozygous and mutant homozygous (hyh) mice. Confocal images of MII oocytes from different genotype after staining cortical granules (CG) with FITC-LCA and DNA with Hoescht 3342. Superficial plane: images were taken at the top surface to observe CG distribution (upper row). CG free domains can be clearly observed. Equatorial plane: images were acquired at the equatorial section in order to see chromatin and its position respect CG free domain of each oocyte (middle row). DIC. Differential interference contrast’s images show cell shape (purple dashed line; lower row). Scale bar: 20 μm.

Cortical granules free domain (CGFD), a meiotic spindle-associated granule-free area, is one of the hallmarks that characterize the asymmetry of cortical granules localization in mouse oocytes. The proposed mechanism for the formation of CGFD is the redistribution of cortical granules during meiotic maturation^5^. Thus, the localization of cortical granules and CGFD (localized over the meiotic spindle) determines two well defined regions in the mouse oocyte (see wild type oocyte in Fig. 7). Surprisingly, when cortical granules (CG) staining was performed in MII oocytes, 9.82% (11/112) of hyh oocytes exhibited an inverted CG distribution: CG were observed over metaphase II spindle and absent in the rest of the cell (Fig. 7). Interestingly, oocytes in which this inverted pattern was observed, showed a less circular shape, and more oval, than the typical circular shape observed for MII oocytes at the equatorial plane (Fig. 7, DIC panel). To compare these shape differences, roundness parameter was calculated as a shape descriptor. Roundness parameter (R), defined as (4 × Area)/(π × Major axis^2^), is a measure of the object shape relative to a perfect circle (R value of 1). As expected, hyh oocytes with atypical distribution of cortical granules presented a significantly lower R value than wild type, heterozygous, and even those hyh oocytes that had a normal distribution of cortical granules (Table 4). In conclusion, only oval-shaped hyh oocytes showed the atypical distribution of cortical granules.

**Table 4:** Oocyte roundness analysis in MII oocytes of wild type, heterozygous and mutant homozygous (hyh) mice.

Genotype	oocyte N°	Roundness
wild type	43	0,980 ± 0,002
heterozygous	29	0,978 ± 0,001
hyh(typical CG distribution)	30	0,976 ± 0,002
hyh(atypical CG distribution)	9	0,905 ± 0,018***

ImageJ’s Roundness parameter calculation was measured using differential interference contrast (DIC) images from MII oocytes of different genotypes. Roundness resulted significantly lower in hyh MII oocytes with atypical CG distribution than in those oocytes with typical CG distribution (wild type, heterozygous and mutant homozygous (hyh); ***p ≤ 0.001; Tukey’s test for multiple comparisons).

### Hyh oocytes have a deficient cortical reaction

**Figure 8:**
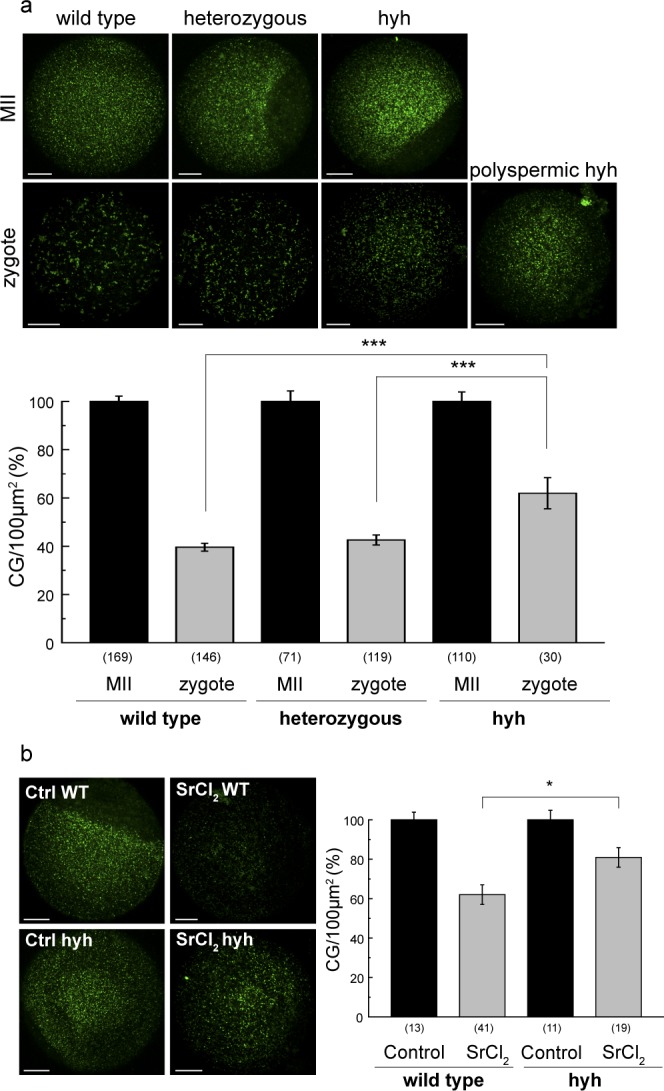
Hyh oocytes exhibit an impaired deficient cortical reaction. (**a**) Cortical granule exocytosis (CGE) after IVF in wild type, heterozygous and hyh oocytes.Upper panel: representative confocal images of MII oocytes and 2PN zygotes of each genotype, stained with FITC-LCA to label cortical granules. A polyspermic hyh zygote is presented to show the absence of CGE despite fertilization. Scale bar: 20 μm. Lower panel: histogram showing CG density/100 μm^2^ for each genotype. Data are shown as mean ± SEM from at least 4 independent experiments. Numbers in parentheses below bars represent the total number of cells analyzed; ***p ≤ 0.001 (Tukey’s test for multiple comparisons, zygotes). Zygotes vs. MII oocytes comparison in each genotype show significant CGE (***p ≤ 0.001; Tukey’s test for multiple comparisons). (**b**) CGE functional assay in wild type and mutant homozygous (hyh) MII oocytes. MII oocytes were subjected to CGE activation triggered with 30 mM strontium chloride (SrCl_2_). Left, representative confocal images of MII oocytes stained with FITC-LCA to label cortical granules. Scale bar: 20 μm. Right, histograms showing CG density/100 μm^2^ for each genotype. Data are shown as mean ± SEM; numbers in parentheses represent the total number of MII oocytes in each condition. *p ≤ 0.05 (Tukey’s test for multiple comparisons). Control vs. SrCl_2_ activated MII oocytes comparison in each genotype show significant CGE (***p ≤ 0.001; Tukey’s test for multiple comparisons).

We have documented that wild type alpha-SNAP mediates the essential process that prevents polyspermy in mouse eggs: the cortical granule exocytosis or cortical reaction^24^. Based on the observation that mutated alpha-SNAP M105I is mislocalized in hyh oocytes, we hypothesized that hyh oocytes had defective cortical granule exocytosis. To evaluate cortical reaction in physiological conditions, oocytes from the three genotypes –wild type, heterozygous and hyh- were *in vitro* fertilized with wild type sperm as described in Materials and Methods. After 6–7 h of co-incubation, embryo were fixed and stained for cortical granule quantification as we previously described^24^. Figure 8 shows that all genotypes were able to exocytose cortical granules after physiological activation by sperm, indicating that in all cases cortical reaction took place (compare MII eggs vs. zygotes for each genotype, p ≤ 0.001). However, hyh embryos showed a significantly reduced cortical reaction when compared to wild type and heterozygous embryos (Fig. 8a, upper panel). Those oocytes containing three or more pronuclei showed an indistinguishable cortical granule pattern from wild type MII oocytes (see polyspermic hyh image in Fig. 8a). Next, to confirm that cortical granule exocytosis was defective in hyh oocytes, we analyzed cortical reaction after parthenogenetic activation. In this case, using our functional assay, oocytes were activated by strontium chloride -a known parthenogenetic activator of mouse oocytes- to analyze cortical granule exocytosis^24,27,53^. Again, as shown in Fig. 8b, cortical reaction triggered by strontium chloride was significantly diminished in hyh oocytes. It has been previously shown that overexpression of alpha-SNAP wild type and/or the addition of exogenous recombinant protein in mouse oocytes^24^ and other cellular models^25,54,55^ provoke inhibitory effects on membrane fusion/exocytosis. Since this effect is highly dependent on the concentration and subcellular localization of alpha-SNAP, we decided to test whether the M105I mutation affects the inhibitory properties of alpha-SNAP overload. Since alpha-SNAP M105I is abnormally distributed in hyh oocytes, we speculated that the inhibitory effect of alpha-SNAP M105I on cortical granule exocytosis would be less dramatic when compared with the effect of the wild type protein. To test this, alpha-SNAP wild type and alpha-SNAP M105I recombinant proteins were microinjected in wild type MII oocytes prior to strontium activation and, then, cortical granule exocytosis was evaluated as described above. Confirming our prediction, results showed that overload of both proteins –wild type and M105I- inhibited significantly cortical granule exocytosis; however, the inhibitory effect was poorer when M105I was microinjected (Supplementary Fig. S1). Altogether, these findings confirmed our hypothesis and indicated that M105I mutation has a negative effect on alpha-SNAP function, leading to defective cortical granule exocytosis.

### Hyh oocytes have a low fertilization rate and a high polyspermy rate

**Figure 9:**
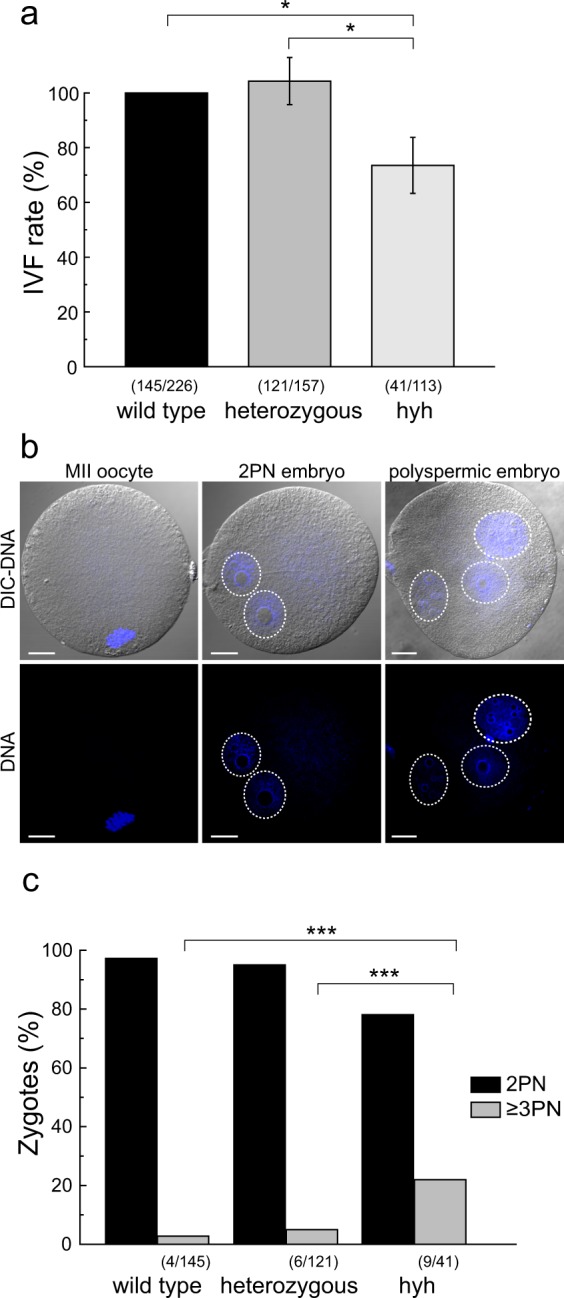
*In vitro* fertilization assays of MII oocytes superovulated from wild type, heterozygous and mutant homozygous (hyh) mouse ovaries. (**a**) *In vitro* fertilization (IVF) rate. MII oocytes from wild type, heterozygous and mutant homozygous (hyh) mice were coincubated with capacitated sperm from wild type mice; fertilization was evaluated after 6–8 h. Data are shown as mean ± SEM from at least 3 independent experiments; numbers in parentheses indicate the number of zygotes on total oocytes analyzed for each genotype assessed. IVF rates are relative to wild type group, set as 100%. *p ≤ 0.05 (Tukey’s test for multiple comparisons). (**b**) Representative confocal images of fixed cells that show the patterns obtained post IVF: unfertilized MII eggs (MII oocyte); normal fertilized egg with two pronuclei (2 PN embryo); polyspermic fertilized eggs that have three or more pronuclei (polyspermic embryo). Blue: DNA labeled with Hoechst 3342; grey: differential interference contrast (DIC) images. Images were taken at DNA confocal plane. Dotted white circles indicate pronuclei. Scale bar: 20 μm. (**c**) Incidence of polyspermy in hyh mice. Quantification of pronuclei in embryos from (**b**). Histogram show 2 PN and ≥3 PN zygotes rate in wild type, heterozygous and mutant homozygous (hyh) embryos obtained from IVF. Numbers in parentheses indicate the number of polyspermic (≥3 PN) zygotes from total analyzed embryos. ***p ≤ 0.001 (chi-squared test). At least 3 independent experiments were performed for each genotype.

We have demonstrated previously that homozygous hyh mutant females are severely subfertile compared to wild type females^42^. Even more, we showed that ovulation rate of hyh superovulated female mice was significantly reduced relative to wild type mice^42^. Based on the finding that alpha-SNAP M105I leads to a deficient cortical reaction, we predicted that hyh oocytes would have polyspermyc fertilization. Therefore, each oocyte genotype -wild type, heterozygous, and hyh oocytes-was coincubated with wild type spermatozoa. After 7 h of coincubation, cells were fixed and fertilization was evaluated by the observation of pronuclei. The results showed that hyh oocytes had a low fertilization rate compared to wild type and heterozygous oocytes (Fig. 9a). When *in vitro* fertilization (IVF) was evaluated, approximately 20% embryos had three or more pronuclei (Fig. 9b,c), which meant that more than one spermatozoon was able to fertilize the oocyte (polyspermy). As shown in Fig. 9c, when polyspermy was assessed after IVF assays, we found that wild type and heterozygous oocytes had 2.75 (4/145) and 4.95% (6/121), respectively of polyspermy, while hyh oocytes had 21,9% (9/41). It is worth to mention that polyspermy (Fig. 9c) was assessed only in those oocytes that were fertilized (Fig. 9a). Interestingly, these results were similar to those informed recently in zygotes resulting from Maternull oocytes, which have established defects in cortical reaction and post-fertilization block^4^.

To demonstrate that hyh MII oocytes were fully competent to achieve meiotic divisions and have no problems in chromosome segregation^12^, we stained fluorescently chromosomes, actin microfilaments and microtubules to analyze chromosomes localization. Results showed that 100% of the analyzed cells had normal alignment of chromosomes on the spindle, there were no spindle abnormalities and only one actin cap over the spindle was observed (Supplementary Fig. S2). In conclusion, and confirming our prediction, these findings show that alpha-SNAP M105I leads to a high polyspermy rate in hyh oocytes. In addition, these results also show that hyh oocytes had a low fertilization rate.

## Discussion

The present study shows that oocytes from hyh female mice exhibit different alterations at cellular and ultrastructural level that adversely affect female fertility. We reported previously that M105I mutation in hyh females plays a critical role in the balance between folliculogenesis and follicular atresia producing a reduced ovulation rate and, consequently, a noticeable reduction of female fertility^42^. Therefore, the pleiotropic phenotype of hyh oocytes described in the present work could result from the effect of the mutated alpha-SNAP in the oocyte’s physiology and from a defective oocyte growth in a non favourable environment.

The fusion of cortical granules with the oocyte plasma membrane –known as cortical granule exocytosis or cortical reaction- is the most significant event to prevent polyspermy. This exocytosis is different from other regulated secretory vesicles because their kinetic is slower and cortical granules are not renewed after their fusion with the plasma membrane^27^. It is accepted that this membrane fusion is driven by the SNARE proteins, which are the minimal machinery for membrane fusion. SNARE complex is disassembled by the joint action of alpha-SNAP and NSF proteins. We have previously documented that alpha-SNAP and NSF participate in cortical granule exocytosis in mouse oocytes. We have proposed a working model in which the alpha-SNAP/NSF complex disassembles cis-SNAREs in a prefusion step^24^. It is worth mentioning that this mechanism would not be valid in sea urchin eggs -where cortical vesicles are docked to plasma membrane^56^- since it has been described that membrane fusion of these secretory vesicles may occur in the absence of NSF^57^. Our study shows that subcellular localization of alpha-SNAP -in both GV and MII hyh oocytes- is particularly altered, displaying a notable change in the localization of the protein from cortex to cytosolic region. The hyh M105I mutation resides in a region of alpha-SNAP that does not interact with the SNARE complex. *In vitro* studies have shown that alpha-SNAP M105I can bind to and disassemble SNARE complexes in a similar way to the wild type alpha-SNAP. These observations, together with the fact that the hyh mice have a diminished amount of alpha-SNAP (hypomorphism) in several tissues, have led to the assumption that the hyh phenotype is due to hypomorphism and not to a malfunction of the mutated protein. Nevertheless, experiments performed by us in hyh sperm (cellular level) indicate that alpha-SNAP M105I has an intrinsic malfunction^58^. These results suggest that M105I mutation may interfere or promote the interaction of alpha-SNAP with cellular components not present in *in vitro* assays, such as membrane lipids or non-SNARE interacting proteins.

Alpha-SNAP and NSF were initially thought to act as soluble factors that transiently bind to SNARE complexes at the moment of membrane fusion, to prime (activate) and/or recycle SNARE proteins. However, evidence obtained from different tissues and cellular models indicates that both alpha-SNAP and NSF are rather membrane-bound proteins^59^. Biochemical experiments performed by Steel and collaborators suggested that alpha-SNAP behaved as a hydrophobic protein able to directly interact with membrane lipids. Furthermore, lipid interaction enhanced the ability of alpha-SNAP to stimulate ATPase activity of NSF^60^. In accordance with these results, Winter and collaborators have identified a conserved hydrophobic loop (membrane-attachment site) within the N-terminal domain of alpha-SNAP^61^. Moreover, they found that alpha-SNAP is functionally less efficient to disassemble SNARE complexes when hydrophobic residues within the loop are mutated or when SNARE proteins are soluble; i.e., lipids are absent during the reaction. Altogether these results indicate that, at steady state, alpha-SNAP is predominantly membrane-bound by direct interaction with membrane lipids. Furthermore, this protein-lipid interaction enhances the functionality of alpha-SNAP noticeably. Our results suggest that M105I alters the subcellular distribution of alpha-SNAP in oocytes. In this regard, the distribution of alpha-SNAP in wild type oocytes is predominantly cortical, probably reflecting its membrane-binding capacity (of cortical granules membrane and/or oolema). However, in mutant oocytes, cortical distribution is reduced and ‘cytosolic’ distribution is increased. Considering that no changes were observed in the number and distribution of cortical granules, the changes observed in alpha-SNAP localization suggest that the M105I mutation may negatively affect protein-lipid binding. Thus, the M105I mutation appears to reduce the functional efficiency of alpha-SNAP in oocytes, preventing proper cortical granule exocytosis and allowing the fusion of more that one sperm (polyspermy) during fertilization. Similarly, the spermatozoa of hyh mice have a reduced capacity to undergo acrosomal reaction, which consists in the exocytosis of their secretory granule, the acrosome^58^. Further studies are needed to demonstrate the impact of M105I mutation, located in the fifth alpha-helical domain, on the hydrophobic loop, located between first and second alpha-helical domains, and on its membrane binding properties^40,41,61^.

One striking observation emerging from this work was the inverted localization pattern of cortical granules observed in almost 10% of oocytes from different females with hyh genotype. This result suggests that alpha-SNAP could be related to the distribution of CG establishment during oocyte maturation. Regarding the role of alpha-SNAP in cellular polarity, although there is not much evidence, it has been reported that in MDCK polarized epithelial cells, alpha-SNAP is involved in the transport from the trans-Golgi network to the apical and basolateral membrane^62,63^. Additionally, alpha-SNAP is required for the distribution of proteins involved in cell-cell contact and it has been characterized that hyh mice have an abnormal localization of several apical proteins in neurons, such as: E-cadherin; α-Catenin; β-Catenin; and Pals1^40^. Alpha-SNAP negatively regulates AMPK activation via LKB1^37^. The fact that LKB1 and AMPK have been implicated in cell polarity establishment^64,65^ raises the possibility that M105I mutation might cause the altered localization of CG in hyh oocyte and other cellular processes like metabolism, cell growth, and development^37^.

Hyh oocytes showed different alterations at ultrastructural level. Under normal circumstances mitochondria are distributed throughout the cytoplasm, but mostly concentrated around the germinal vesicle in the GV stage and surrounding the meiotic spindle in the metaphase II oocytes^66,67^. This pattern of distribution was observed in wild type but not in hyh oocytes, in which mitochondria were uniformly dispersed throughout the cytoplasm (Supplementary Fig. S3). The ultrastructural analysis of wild type and hyh oocytes showed normal general morphology of most organelles, except for mitochondria. Electron micrographs showed that mitochondrial diameter and vacuole size were significantly decreased in hyh compared to wild type MII oocytes. These observations suggest that mitochondria function may be altered. Interestingly, it has been demonstrated that alpha-SNAP regulates mitochondrial biogenesis^37^. In addition, mitochondrial disfunction has been associated with the release of extracelular material in aged cells^68^.

On the other hand, an accumulation of dense lamellar bodies and phagophore-like structures at different stages of maturation was observed in hyh oocytes. This observation is consistent with the function of alpha-SNAP in the maturation of autophagosome reported by Abada and collaborators^44^. This group demonstrated that the knockdown of alpha-SNAP leads to inhibition of autophagy, manifested by an accumulation of autophagosomes located in close proximity to lysosomes but not fused with them. On the other hand, it has been proposed that loss of alpha-SNAP induces non-canonical autophagy in human epithelia^30^. Even more striking, numerous extracellular vesicles, cytoplasmic remnants, and membranous debris were observed exclusively in the perivitelline space of hyh oocytes. The secretion of these structures could be consistent with different processes such as exosome release^69^, exocytosis of residual bodies, or autophagic exocytosis^52,70^. These findings have been described as evidence of degenerative changes related to apoptotic processes^71^ or oocyte aging^72,73^; and the secretion of this type of structures is a consequence of the accumulation of autophagic or degradative vesicles in the cytoplasm. Our results suggest that in hyh oocytes, degradative pathways could be increased or, alternatively, impaired, with a consequent accumulation of degradative products in the cytoplasm and perivitelline space. Hence, these observations indicate that alpha-SNAP is required to prevent this abnormal accumulation of degradative structures.

The interplay between autophagy and apoptosis is now beginning to be understood as a result of the discovery of a dialogue between the mediators of the molecular machinery of autophagy and programmed cell death. In this sense, it has been reported that alpha-SNAP promotes autophagic flow in epithelial cells^34^ and that it is critical to generate anti-apoptotic defenses due to its direct interaction with the proapoptotic protein BNIP1^31^ or by modulating Bcl-2 expression and thus its prosurvival signaling^32^. We have previously reported that alpha-SNAP plays a significant role in the balance between ovarian follicular development and atresia, since hyh mutant mice showed an increased rate of apoptosis in granulosa cells, cumulus cells and follicular atresia^42^. A high rate of apoptosis in hyh granulosa and cumulus cells accounts for possible interference in the bidirectional communication between oocyte and their partners’ somatic cells, which is essential for normal follicular development and differentiation, as well as the acquisition of the oocyte developmental competence to undergo successful fertilization and support embryogenesis^74,75^. In this sense, similar alterations in this interplay in homozygous female carriers of M105I mutation could interfere with oocyte quality, its potential to be fertilized and even have an impact on embryonic and fetal development.

Here we also report a diminished *in vitro* fertilization rate of hyh oocytes compared to wild type and heterozygotes controls, which demonstrate a decreased oocyte ability to interact with the sperm. As in the case of hyh spermatozoa^58^, the difference between genotypes was less dramatic in IVF assays than in our previous mating studies^42^. These results suggest that there are others additional factors participating during the interaction between the oocyte and the sperm during fertilization. In addition, we documented that oocytes from hyh female mice have a high polyspermy rate. Interestingly, it has been documented that postovulatory aged eggs show a reduced ability to support sperm-egg interaction (low fertilization rate) and a reduced ability to establish the membrane block to polyspermy (high polyspermy rate)^76–78^. Similarly, it has been documented that aged oocytes may exhibit an atypical distribution of cortical granules over the meiotic spindle^79^. From these similarities, we might speculate that hyh MII oocytes have aging-like features.

In summary, our findings indicate that the M105I mutation in alpha-SNAP leads to a dramatic decrease in fertility due to severe multifactorial defects in hyh oocytes, including mislocalization of alpha-SNAP M105I, a striking accumulation and secretion of degradative structures, and an atypical distribution of cortical granules in oval MII oocytes. In addition to these structural observations, hyh oocytes showed functional alterations such as a low fertilization rate, a deficient cortical reaction and a high polyspermy rate. It remains to be explored if these changes are either a cascade of changes or separate processes triggered by the M105I mutation in the alpha-SNAP protein. Performing an oocyte-specific conditional alpha-SNAP M105I mutant mouse to analyze the oocyte growth in a context of wild-type folliculogenesis would allow clarifying this point; nevertheless, this specific conditional mouse has not been created so far.

## Methods

### Reagents

All chemicals, unless stated otherwise, were purchased from Sigma-Aldrich Chemical Inc. (St. Louis, USA).

### Animals, superovulation, and oocyte collection

This study was carried out in strict accordance with the recommendations of the Guide for the Care and Use of Laboratory Animals of the National Institutes of Health. The protocol was approved by the Institutional Animal Care and Use Committee of the Universidad Austral de Chile. Wild type (Napa(+/+)), mutant heterozygous (Napa(+/M105I)) and homozygous (Napa (M105I/M105I; called hyh) mice were obtained from The Jackson Laboratory (Bar Harbor, ME, USA)^80^. These animals were bred into a colony at Facultad de Medicina, Universidad Austral de Chile, Valdivia, Chile. All animals were genotyped by a PCR-based method^81^. This study included only adult animals (8–20 weeks). Mice were fed ad libitum with rodent food and kept under a constant photoperiod of light/dark 12:12 h and room temperature of 22 °C.

### Oocyte collection

Oocyte collection was performed as described in our previous work^24^. GV oocytes were obtained from females primed by intraperitoneal (i.p.) injections with 10 IU of pregnant mare’s serum gonadotropin, PMSG (Syntex, Argentina), and 45–48 h later cumulus-oocyte complex were obtained by puncturing ovarian follicles. The collection medium was Earle’s balanced salt solution with 0.01% PVA, 0,001% Gentamycin, and 25 mM Hepes buffer, pH 7.3 (MEM/HEPES) supplemented with 2.5 μMMilrinone to inhibit oocyte maturation. Only GV oocytes about 80 μm in diameter and intact cumulus were used. After being pipetted repeatedly through a thin-bore pipette, cumulus cells were removed. MII oocytes were obtained from females primed with 10 IU i.p. of pregnant mare’s serum gonadotropin (PMSG; Syntex, Argentina) followed by 10 IU i.p. of human chorionic gonadotropin (hCG (Syntex, Argentina) 48 hr later. MII oocytes were collected from the oviductal ampullae by the scratching method between 13–17 h after hCG injection into MEM/HEPES, and denuded of cumulus cells by brief exposure to 0.04% hyaluronidase. Both GV oocytes and MII oocytes were cultured until use in drops of CZB medium (EmbryoMax® CZB, Merck Millipore) under mineral oil at 37 °C in a humidified atmosphere of 5% CO_2_ in air.

### *In vitro* fertilization

*In vitro* fertilization was performed as described in our previous work^24^. For *in vitro* fertilization sperm culture, as well as gamete coincubation, was carried out into human tubal fluid (HTF) containing 5 mg/ml BSA covered with mineral oil, at 37 °C in a humidified atmosphere of 5% CO_2_ in air. Spermatozoa were obtained from adult male mice (3–6 months old) with proven fertility by excising the cauda epididymis. After incubation for 15 min, the sperm concentration was adjusted at 5 × 10^6^ sperm/ml and incubated for 2 h in HTF for capacitation. For coincubation, the sperm suspension was diluted to obtain 100 μl insemination drops containing 1–5 × 10^5^ sperm/ml. MII oocytes were collected from superovulated females as described above. 10–20 MII oocytes were incubated in each drop. After 6–7 hs of insemination, oocytes were washed using a thin-bore pipette to remove loosely attached sperm before 1 h fixation in 4% PAF. *In vitro* fertilization was evaluated by observation of female and male pronuclei.

### Artificial activation of Metaphase II oocytes

After collection, MII oocytes were thoroughly washed in calcium/magnesium-free CZB, and activated by freshly prepared SrCl_2_ 30 mM in the same medium, during 1 h as we described previously^24^. All incubations were performed at 37 °C in a humidified atmosphere of CO_2_ (5%) in air. After activation, oocytes were immediately processed for cortical granule staining.

### Cortical granule staining and quantification

Cortical granule staining and quantification were performed as previously described^24^. The zona pellucida was removed by brief incubation in acid Tyrodes, pH 2.2, washed in MEM/HEPES and fixed in 3.7% paraformaldehyde in Dulbecco’s PBS (DPBS) for 1 h at RT. After fixation, cells were washed in blocking solution (BS) containing 3 mg/ml BSA, 100 mM glycine and 0,01% Tween 20 in DPBS before permeabilization with 0.1% Triton X-100 in DPBS for 15 min. After 3 washes in BS, cells were incubated in 25 μg/ml FITC-LCA in BS for 30 min, and mounted in Vectashield MountingMedium (Vector Laboratories, Burlingame, CA) containing 1.5 μg/ml Hoechst 33342 (Molecular Probes, Invitrogen) for DNA labeling on a slide, sealed, and stored at 4 °C until visualization. The images on flat optical fields of cortex resulting from partial compression of cells by the coverslip were acquired with a confocal laser-scanning microscope (FV1000, Olympus) using a PLAPON 60x/NA1.42 oil-immersion objective lens, at 512 × 512 pixel resolution. The confocal acquisition parameters remained constant for all captured images within the same experiment. The cortical granules density per 100 μm^2^ (CG/100 μm^2^) for each cell was determined as the mean of the counts from at least four non overlapping equal areas of cortex containing cortical granules by computer-assisted image quantification using ImageJ. For each group, relative CG density/100 μm^2^ was calculated by comparing the mean density of CGs of the treated group with the mean density of CGs of the untreated control group, according to the following equation: [density of CGs in treated group/density of CGs in untreated group] x100, thus setting density of CGs in untreated group (control condition) as 100%.

### Immunofluorescence

Oocytes were briefly exposed to acidic Tyrode’s solution pH 2.2, to remove the zona pellucida and fixed in 3,7% paraformaldehyde in DPBS for 1 h at RT as we previously described^24^. Fixed cells were washed in BS and permeabilized with 0.1% Triton-X in DPBS for 15 min at RT. Following permeabilization, oocytes were washed 3 times in BS and incubated with primary antibodies diluted in BS overnight at 4 °C, at the indicated final concentrations: monoclonal anti-α-/β-SNAP antibody 50 ng/μl (Synaptic Systems, clone 77.2) and rabbit polyclonal anti-NSF antibody 1:20 dilution, (Synaptic Systems). After washing, cells were incubated with the secondary antibody at RT for 1 h. The secondary antibodies used were: donkey anti-mouse, DyLight 488 conjugate (5 ng/μl, Jackson InmunoReasearch) to detect α SNAP; goat anti-rabbit, Alexa Fluor 594 conjugate (5 ng/μl, Thermo Fisher) to detect NSF. After washing, cells were mounted in Vectashield Mounting Medium (Vector Laboratories, Burlingame, CA) containing 1.5 μg/ml Hoechst 33342 (Molecular Probes, Invitrogen) for DNA detection on a slide under minimal compression, sealed, and stored at 4 °C until visualization. Nonspecific staining was determined by incubation without primary antibody. Images were obtained at the equatorial region of the cells using a FV1000 Confocal Microscope (Olympus), with a PLAPON 60x/NA1.42 oil-immersion objective lens, at 512 × 512 pixel resolution. For each experimental series, images were captured using the same microscope settings. ImageJ software was used for the analysis of the images.

### Transmission electron microscopy

The oocytes were first fixed in 2% paraformaldehyde in DPBS for 15 min at RT and briefly washed 3 times with DPBS. Then they were placed in the bottom of a thin-walled 0.2 ml tube with a minimum amount of medium, covered with 100 µl agarose at 40 °C and centrifuged 2 min at 700 x g. The tubes were placed in ice to allow the solidification of agarose. The agarose plugs with the oocytes were removed using ice-cold water in a syringe. Under a dissecting microscope, the excess of agarose was trimmed off, and the plugs were placed in 1 ml of primary fixative (2.5% glutaraldehyde; 4% PAF; 0.1% tannic acid; 0.01 M MgCl2, pH 7.4) for 3 h at RT with orbital agitation. Primary fixation was followed by 4 washes for 15 min with cold PBS. A secondary fixation was performed with 1% osmium tetroxide (Ted Pella, Inc) at 4 °C. Samples were then washed 3 times with cold PBS for 15 min and two additional washes with cold Milli-Q water for 10 min. All washing steps were performed with gentle agitation. The samples were then dehydrated through a series of graded acetone (Merck) (on ice: 30%, 50% and 70%; at RT: 80, 95%, and three final dehydration steps with 100% acetone). All dehydration steps were performed for 15 min. The samples were then embedded in 1:1 acetone:Epon resin (Ted Pella, Inc.) overnight at RT and finally placed in embedding BEEM capsules with fresh 100% Epon resin (Ted Pella, Inc.) overnight at RT. Samples were cured for 36 h at 60 °C in oven (M19, THELCO), and processed at an Academic Facility (Servicio Central de Microscopía Electrónica de la Facultad de Ciencias Veterinarias, UNLP, Argentina) where ultra-thin sections were cut using a diamond knife on a Leica EM Ultramicrotome (Leica, Vienna, Austria), contrasted with 1% uranyl acetate and Reynolds solution. A JEM 1200 EX II electron microscope (JEOL, Tokio, Japan) was utilized for examination and images were recorded on Erlangshen ES1000W 785 digital camera (Gatan Inc., Pleasanton, California, USA). This protocol was modified from Anguish & Coonrod 2013. For wild type and hyh (three mice for each genotype) MII stage oocytes, 5–7 oocytes were analyzed and 30–50 micrographs from nonadjacent sections were obtained. The mean size calculation resulted from a mean of major axis measure for the different analyzed ultrastructures. According to Abbott *et al*. 2001, the distance of each CG to the plasma membrane was determined by taking the shortest perpendicular measurement from the plasma membrane to the CG surface closest to the plasma membrane.

### Data analysis

Data are presented as mean ± SEM, unless otherwise indicated. The number of oocytes used for each experiment is indicated in the figure legends. Data analysis was performed using KyPlot software. Statistical significance was determined by Student’s t-test, or One-Way Analysis of Variance (ANOVA) followed by Tukey’s test for multiple comparisons. Statistical comparisons in frequency histograms were performed using the Kolmogorov-Smirnov test for two sets of data. In all cases, p < 0.05 was considered statistically significant.

## Supplementary information

Dataset 1
