# Supplementary material for: Pleiotropic effects of alpha-SNAP M105I mutation on oocyte biology: ultrastructural and cellular changes that adversely affect female fertility in mice
Source: Sci Rep. 2019 Nov 22;9:17374. doi: 10.1038/s41598-019-53574-8 (PMC6874563; doi:10.1038/s41598-019-53574-8)
Supplement: Supplementary file 1 — Dataset 1 [file 41598_2019_53574_MOESM1_ESM.pdf]

Supplementary Material

Title:

Pleiotropic effects of alpha-SNAP M105I mutation on oocyte biology: ultrastructural and cellular changes that adversely affect female fertility in mice

Auhors:

Matilde de Paola, María Paz Miró, Marcelo Ratto, Luis Federico Bátiz, Marcela Alejandra Michaut

Supplementary Figure S1

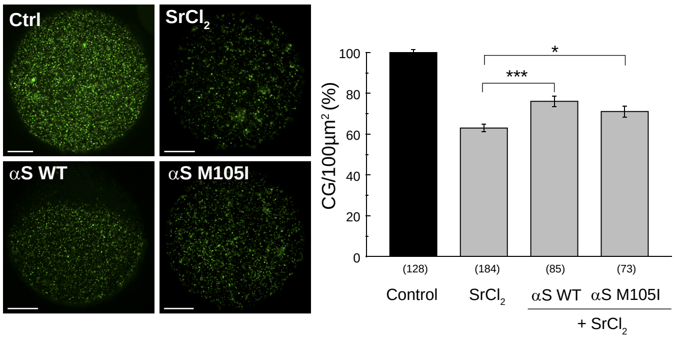

Supplementary Figure 1. Effect of recombinant mutant α-SNAP M105 on CGE. MII oocytes were microinjected with wild type α-SNAP (αS WT) or mutant α-SNAP M105I (α S M105I) prior to CGE activation with 30 mM strontium chloride (SrCl2). Left, representative images of MII oocytes stained with FITC-LCA to label cortical granules. Scale bar: 20 µm. Right, histogram showing %CG density/100 µm2 relative to untreated group (Control) set as 100%. Data are shown as mean ± SEM from at least 5 independent experiments; numbers in parentheses represent total number of MII oocytes. *** $p \leq 0.001$; * $p \leq 0,01$ (Tukey's test for multiple comparisons).

Supplementary Figure S2

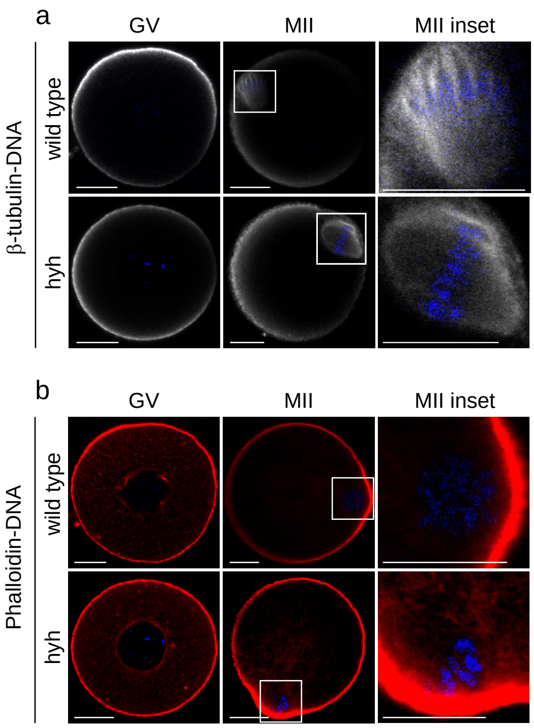

Supplementary Figure 2. β-tubulin localization and F-actin staining during meiotic maturation in wild type and mutant homozygous (hyh) oocytes. a. β-tubulin was immunodetected at two different stages during meiotic maturation: GV-intact oocytes (GV) and MII oocytes (MII). MII Inset: β-Tubulin immunostaining of the metaphase II spindle in the same MII oocyte. Grey indicates positive staining for primary β-tubulin antibody, detected by a secondary antibody conjugated to Alexa Fluor 635; blue indicates DNA, labeled with Hoechst 3342. Scale bar: 20 µm. b. Representative confocal images of F-actin staining pattern at two different stages during meiotic maturation: GV-intact oocytes (GV) and MII oocytes (MII). MII Inset: Cortical F-actin cap in the domain adjacent to the metaphase II spindle in the same MII oocyte. F-actin was detected by using phalloidin conjugated to rhodamine (red). DNA was labeled with Hoechst 3342 (blue).Scale bar: 20 µm.

Supplementary Figure S3

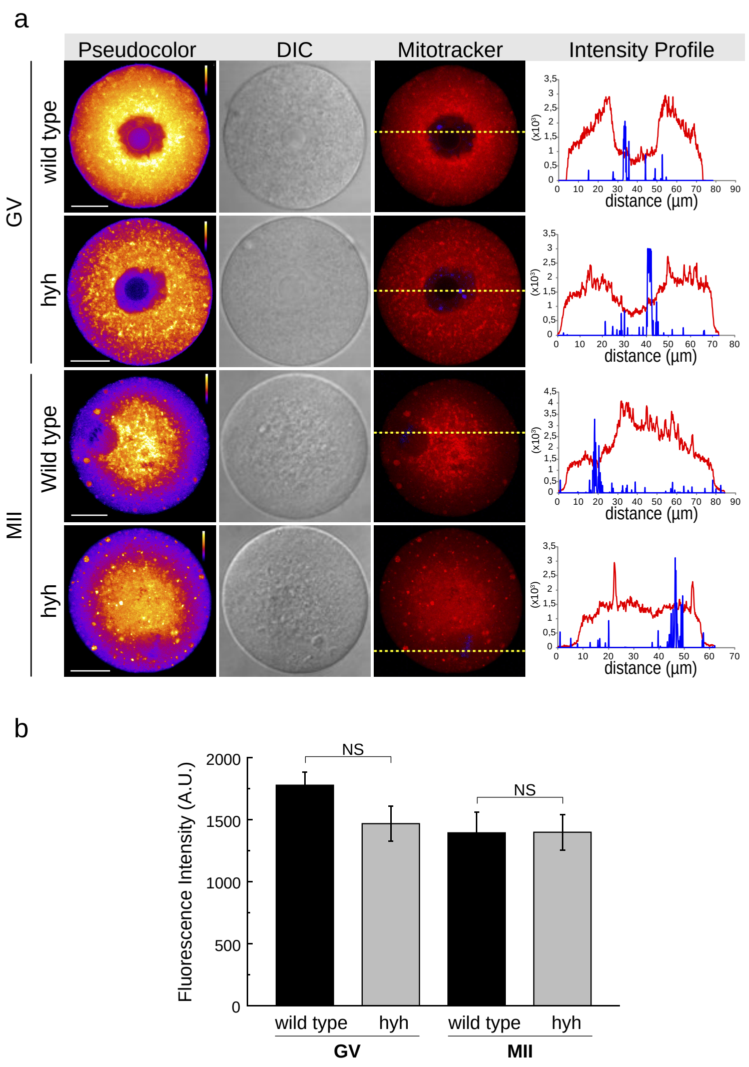

Supplementary Figure 3. Mitochondrial analysis during meiotic maturation in wild type and mutant homozygous (hyh) oocytes. Representative confocal images of GV-intact oocytes (GV) and MII oocytes (MII) in wild type (a) and mutant homozygous (hyh) oocytes (b), after staining with Mitotracker Orange CM-H2TMRos to label mitochondria, and Hoescht 3342 to detect DNA. DIC shows differential interference contrast images. Images were taken at equatorial section to detect chromatin. Left column in each panel shows pseudocolor image processing of each oocyte. Calibration bar shows relative fluorescence intensity (0, blue: minimum intensity; 4095, white: maximum intensity). Right column in each panel shows the fluorescence intensity profiles for Mitotracker (red line) and Hoescht 3342 (blue line). Fluorescence intensities were measured along dashed yellow lines traced in each oocyte, comprising DNA. Scale bar: 20 µm. c. Histogram showing flourescence intensity quantification for wild type MII oocytes compared to hyh in GV and MII oocytes. N.S $p>0.05$ (Student's t-test). Number of analyzed cells: GV wild type=21; GV hyh=19; MII wild type MII =14; MII hyh=7.

Supplementary Materials and Methods

β-tubulin immunofluorescence

Immunofluorescence was performed as previously described in Materials and Methods. Mouse monoclonal anti-β-tubulin (1:200 dilution, Sigma-Aldrich, clone TUB 2.1) was used as primary antibody. Alexa fluor 633 conjugate (4 ng/µl, Thermo Fisher) was used as secondary antibody to detect β-tubulin.

Microfilament and mitochondrial staining

F-actin detection was performed using Rhodamine Phalloidin (1:400 dilution, Molecular probes, Invitrogen) for 1 h at 37 ºC after cell fixation and permeabilization as described for immunofluorescence. Mitochondrial staining was carried out by incubating oocytes in CZB medium containing 2,5 µM Mitotracker Orange CMH2TMRos (Molecular Probes, Invitrogen) for 40 min at 37 ºC, and then fixed as previously described. After microfilament and mitochondrial staining, cells were finally washed in BS and mounted in Vectashield Mounting Medium (Vector Laboratories, Burlingame, CA) containing 1.5 µg/ml Hoechst 33342 (Molecular Probes, Invitrogen) for DNA detection, on a slide under minimal compression, sealed, and stored at 4°C until visualization. Images were obtained using a FV1000 Confocal Microscope (Olympus), with a PLAPON 60x/NA1.42 oil-immersion objective lens, at 512 x 512 pixel resolution. For each experimental series, images were captured using the same microscope settings. Confocal images were analyzed and edited using ImageJ software (version 1.42l; NIH, MD).
